# Identification of sleep fragmentation-induced gut microbiota alteration and prediction of functional impact in Sprague Dawley rats harboring microbiome derived from multiple human donors

**DOI:** 10.5935/1984-0063.20200116

**Published:** 2022

**Authors:** Judy Triplett, Amber Braddock, Erin Roberts, David Ellis, Victor Chan

**Affiliations:** 1 Oak Ridge Institute for Science and Education, Air Force Research Laboratory, 711th Human Performance Wing - Oak Ridge - TN - United States.; 2 Henry M. Jackson Foundation for the Advancement of Military Medicine, Air Force Research Laboratory, 711th Human Performance Wing - Wright-Patterson Air Force Base - OH - United States.; 3 Air Force Research Laboratory/711 HPW, Cognitive Neuroscience Section, Performance Optimization Branch, Airman Systems Directorate - Wright Patterson Air Force Base - OH - United States.

**Keywords:** Sleep Fragmentation, Sleep Disturbances, Gut Microbiota, Humanized Rat Model, Metagenomics, 16S rRNA

## Abstract

**Objectives:**

Poor quality sleep, including sleep fragmentation (SF), can result in severe health consequences. Gut microbiota symbiotically coexist with the host, making essential contributions to overall well-being. In this study, the effects of both acute (6-day) and chronic (6-week) SF in a humanized rat model were examined to evaluate the impact of SF on this symbiotic relationship.

**Material and Methods:**

Human fecal material was transplanted into antibiotic-treated, microbially depleted, Sprague Dawley rats. Animals were subjected to either acute or chronic SF and shifts to gut microbiota were investigated using 16S rRNA sequencing and predictive functional profiles were constructed with PICRUSt. We also investigated SF-induced intestinal microbial adhesion and penetration or increased microbial invasion of selected tissues and organs; as well as changes in crypt/villi architecture.

**Results:**

Microbiota profiling indicated that chronic, but not acute, SF significantly decreased the richness of alpha-diversity of distal ileum microbiota, and altered cecum and distal ileum beta-diversity; although both acute and chronic SF significantly changed select populations of microbiota in all three regions. Neither acute nor chronic SF induced changes to microbial adhesion, penetration, or invasion into intestinal tissues or nearby organs. Additionally, we found that chronic SF caused a reduction in villus height in the proximal colon.

**Discussion:**

Our study suggests that acute SF alters the gut microbiota in this humanized rat model, while chronic SF produces more pronounced changes to microbiota populations. This study identified potential microbiota targets for the prevention and/or intervention of the adverse effects of S F.

## INTRODUCTION

Consistent, quality sleep is vital to peak performance. Disrupted sleep not only leads to impaired physical function, mood, and cognition, but also induces stress that may alter the structure and function of gut microbiota communities, creating dysbiosis, a disequilibrium of commensal gut microbes^1,2,3,4,5,6,7,8,9,10^. For example, mice subjected to a brief period (5h) of sleep deprivation led to altered abundance of *Clostridiaceae* and *Lachnospiraceae* compared to controls^2^, while a longer period (5 days) of sleep disruption resulted in an increased *Firmicutes*:*Bacteroidetes* ratio, along with decreased abundance of *Lactobacillus, Actinobacteria*, and *Bifidobacterium*^9^. Rats undergoing 8h of sleep fragmentation for 28 days however showed a lower *Firmicutes*:*Bacteroidetes* ratio, with a reduced abundance of putative short chain fatty acid-producing bacteria and higher *Proteobacteria*^8^. By sampling the gut microbiota from different regions of the intestinal tract after acute and chronic sleep fragmentation treatments, we identified temporal and region-specific changes in rats in our previous study^10^. There were significant perturbations in the alpha- and beta-diversity, especially in the distal ileum, with several taxa (including *Lachnospiraceae, Lactobacillaceae, Ruminococcaceae, Prevotella*, and *Turicibacteraceae,* among others) showing altered abundancies in multiple regions after either/both acute and chronic treatments. Similar to the result of Bowers et al. (2020)^9^, human subjects showed an increased *Firmicutes*:*Bacteroidetes* ratio after two days of partial sleep deprivation^7^, with changes in the abundances of the *Coriobacteriaceae, Erysipelotrichaceae,* and *Tenericutes*.

Dysbiosis of gut microbiota can significantly impact host systems, such as metabolism and immune response, which can lead to degraded host physiology and health^4,5,6^. To illustrate this complex relationship, in a study of nine, healthy men, just two nights of partial sleep deprivation led to decreased insulin sensitivity, a decreased presence of beneficial microbiota, and an altered gut microbiota composition specifically associated with obesity and type 2 diabetes^7^. Further, sleep fragmentation (SF), or repeatedly interrupted sleep without a reduction in total sleep time, which commonly occurs in disorders like sleep apnea, restless leg syndrome or chronic pain, has also been reported to alter microbiota populations and negatively affect health^8,9,10^. And in older adults where SF is more common, links between poor sleep, altered gut microbiome composition and age-related cognitive decline as seen in neurodegenerative conditions such as Alzheimer’s or Parkinson’s have been reported^11^.

Bidirectional communication between the gut microbiota and the brain, often referred to as the microbiome-gut-brain axis, is thought to be a key regulator of human health, as gut microbiota can interact directly or indirectly with the nervous, endocrine, metabolic, and immune systems^12,13^. This gut microbiota-host signaling is vital to maintain healthy function, and dysfunction of this communication is believed to be an important contributing factor in many diseases^14^. Therefore, prebiotic- and/or probiotic-based manipulation of this symbiotic ecosystem may hold great potential for uncovering preventive and/or therapeutic intervention strategies.

Previous studies examining the effects of various sleep disturbances upon gut microbiota have generated mixed results. While most showed that irregular sleep leads to stress-induced microbiota dysbiosis in humans and animals, several studies reported limited perturbations of gut microbiota^7,15,16,17^. These differences may be due to dissimilarities in type of sleep disturbance, time points of sample collections, and/or methodologies used to generate and analyze experimental data. Human subjects, in particular, present challenges in microbiome studies as obtaining region-specific microbiota samples and histological examination of tissues are excessively invasive. One way to address these difficulties involves utilization of animal models harboring solely human gut microbiota via fecal transplant (i.e., ‘humanized’ models that are devoid of native gut microbiota). Multiple humanized models have been developed, each with its own advantages and disadvantages^18,19,20^. While we and other investigators have previously reported dysbiosis of gut microbiota in rats under sleep fragmentation^8,10^, it is not clear if these findings are relevant to humans under the same situation, since humans and rats have very different gut microbiota profiles.

In an effort towards obtaining human-relatable data as to the effect of sleep fragmentation (SF) upon gut microbiota, we conducted both acute (6-day) and chronic (6-week) SF experiments in rats harboring the human gut microbiota from multiple human donors. Intestinal content was collected from three gastrointestinal tract locations (distal ileum, cecum, and proximal colon) following acute or chronic SF, and 16S rRNA gene sequencing performed to identify significant changes in microbiota populations. The functional impact of these changes was inferred using the software tool, PICRUSt. Further, we determined whether SF encouraged intestinal bacterial translocation by evaluating microbial adhesion, penetration, and invasion of surrounding tissues. Finally, we inspected architectural changes in the intestinal crypt/villi of the corresponding regions of the gastrointestinal tract used in microbiota analysis.

## MATERIAL AND METHODS

### Ethics statement

This study protocol was reviewed and sanctioned by the Wright-Patterson Air Force Base Institute of Research, Institutional Animal Care and Use Committee (IACUC), and the U.S. Air Force Surgeon General’s Office of Research Oversight and Compliance. All experiments were performed in compliance with the animal welfare act. All experiments were in compliance with the principles set forth in the “guide for the care and use of laboratory animals”^21^. All work was performed in an Association for the Assessment and Accreditation of Laboratory Animal Care (AAALAC) - accredited facility.

### Methods

**Animals:** Sprague Dawley (SD) rats (specific pathogen free) were acquired from Charles River (2-week old, Wilmington, MA, USA). Initially, all rats were housed in standard, clear plastic cages (2 animals per container) with standard bedding (CellZorb, Cincinnati Lab Supply). Rats had *ad libitum* access to food (LabDiet Formulab Diet 5008, Cincinnati Lab Supply) and water. Rats, when housed in semi-rigid isolators (Charles River), were singly held in sterile cages containing sterile bedding (Alpha Dri Irradiated Bedding, Charles River) located in a germ-free room. Sterile food (Picolab Rodent Irradiated Chow, Charles River) and water was provided *ad libitum*. Rats were maintained in a 12h light/dark cycle at 22°C.

**Fecal transplantation into microbially depleted rats:** the gut microbiota of SD rats was humanized as described previously^22^. Briefly, animals were segregated into semi-rigid isolators (Charles River) and exposed to a 12-day antibiotic treatment (enrofoxacin 200mg/L, neomycin 800mg/mL, and vancomycin 850mg/L), prepared fresh daily in the drinking water. Agar culture of fecal and cecal contents and gel electrophoresis of bacterial 16s rRNA PCR products (collected from antibiotic-treated rats) were examined to ensure that gut microbiota were significantly knocked down prior to fecal transplant. Identical amounts of human fecal material from 5 donors, supplied by OpenBiome (Somerville, MA), were homogenized, mixed in Luria broth (total colony forming unit of 2.04E+09), and introduced to the intestinal tracts of the rats by oral gavage. Animals were inoculated with the human fecal mixture at five time points, on days 1, 3, 5, 8, and 11 (day 1 = the first fecal inoculation), and SF experiments began 2 weeks after the final inoculation. This current study utilized fecal samples from 5 of the 6 donors listed in the previous animal model development study as one subject was excluded from this study due to insufficient fecal material^22^.

**Sleep fragmentation:** *naïve* and humanized rats were individually housed in sleep fragmentation chambers (Lafayette Instrument Company; Lafayette, IN; model No. 80391) where a bar swept horizontally across the cage foor, as described previously by Ramesh et al. (2009)^23^. While this procedure significantly reduced both slow wave sleep and rapid eye movement sleep in mice (based on EEG measurements), it is relatively stress-free in that no increase in the serum level of corticosterone or body temperature was detected in the treated animals^23^. This moving obstacle would awaken the rat, as the rat had to step over the sweeper bar. In the acute SF study, the bar swept across the cage bottom every 3min for 6 days. For the 6-week period of the chronic SF study, the sweeper bar remained stationary for 3 hours each day (12 p.m. to 3 p.m.) for safety reasons, as this longer study would be more strenuous for the animals. The control group (normal sleep) were also housed in the SF chambers under identical conditions, with the exception that the sweeper bar was immobile.

**Sample acquisition, DNA isolation, and library preparation:** after 6 days or 6 weeks of SF, animals were asphyxiated by CO_2_. The liver, spleen, and intestinal material (from the distal ileum, cecum, and proximal colon) were harvested, and tissues from the intestinal walls, mesentery, and mesenteric lymph nodes were collected. All samples were fash frozen in liquid nitrogen, and stored at -80°C, with the exception of the intestinal tissues, which were placed on ice for immediate investigation into bacterial adhesion, penetration and invasion (see below). QIAamp DNA Stool Mini Kit (Qiagen) was utilized to isolate DNA from 250mg of intestinal material. DNA was quantified with a NanoDrop ND-1000 Spectrophotomer (Fisher Scientific). The Ion 16S Metagenomics kit (Fisher Scientific) was used for DNA library preparation for the Ion Torrent system. This kit amplifies a wide range of variable DNA regions, including: V2, V3, V4, V67, V8, and V9 (data from all variable regions were used in OTU identification). Agencourt AMPure XP magnetic beads (Fisher Scientific) were used for DNA purification and an Agilent 4200 TapeStation (D1000) was used for quantification. Ion Plus Fragment Library Kit (Fisher Scientific) was used for DNA end repair and ligation of adapters to 50ng of DNA, which was then labeled with Ion Xpress Barcode Adapters 1-96 Kit (Fisher Scientific).

**DNA sequencing and taxonomic analysis:** sample DNA were pooled and loaded onto 530 chips (Life Technologies) using the Ion Torrent Ion Chef System (Life Technologies). DNA was sequenced on an Ion Torrent S5. DNA processing and quality filtering (Q<10, 400 base pairs) was automated using Ion Torrent Software (Suite v.5.2). Quantitative Insight into Microbial Ecology (QIIME2, v.2018.11) was used to analyse FASTQ files^24^. DNA (250 base pairs) were filtered with a Phred Quality score of 20^25,26^. GreenGenes reference database (v.13.8, 99% sequence identity) was used to assign operational taxonomic units (OTUs)^27^. Alpha diversity was evaluated using Faith’s phylogenetic diversity index and the Kruskal-Wallis test (*p*<0.05); while beta-diversity profiles were gaged by weighted normalized UniFrac and Bray-Curtis methodologies (PERMANOVA, *p*<0.05, 999 permutations) and visualized with Emperor software^28,29,30^. Perturbations to individual microbiota populations were evaluated using Gneiss balance correlation clustering (Ward’s hierarchical methodology)^31^. This analysis does not directly compare relative abundance between taxa as that information is likely biased due to limitations in PCR amplicon production and the compositional nature of sequencing data, which reports abundance in a relative manner. Instead, Gneiss analyses the relative, differential abundance of taxons to characterize niche differentiation.

**PICRUSt analysis:** microbial community functionality was assessed using Phylogenetic Investigation of Communities by Reconstruction of Unobserved States (PICRUSt, v.1.1.3)^32^. OTUs were assigned by 97% sequence identity using a closed reference search against GreenGenes database (v.13.5). OTU tables were normalized and referenced against the Kyoto Encyclopedia of Genes and Genomes (KEGG) Orthology (KO) database. Predictive PICRUSt data were analyzed for statistical significance using Statistical Analysis of Taxonomic and Functional Profiles (STAMP, v.2.1.3) and White’s non-parametric t-test with Benjamini-Hochberg FDR *post hoc* test^33^.

**Bacterial adhesion, penetration, and invasion:** analysis of bacterial adhesion, penetration, and invasion was performed as described previously^10^. Briefly, non-adhered microbial cells were collected from intestinal tissue after a 15min gentle shake with 1mM dithiothreitol in PBS. Adhered microbial cells were isolated after 60 seconds of vigorous vortexing of the tissue with PBS. Penetrated microbial cells were recovered from the washed tissues after homogenization with PBS in a Bullet Blender Gold (Next Advance). Cells were pelleted and stored in 15% glycerol in lysogeny broth. Microbial titers (from adhesion and penetration isolation, intestinal content or homogenized organ tissue) were determined by aerobic and anaerobic culturing on 5% sheep blood agar plates with TSA (Fisher Scientific, ran in duplicate). BD GasPak EZ Gas Generating System (Fisher Scientific) was use in the culturing of anaerobic bacterial colonies and a Scan 300 Automatic Colony Counter (Interscience) tallied colony expansion to calculate colony-forming units (CFU) per gram of tissue/material.

**Hematoxylin and eosin staining of intestinal tissue:** sliced intestinal tissues (12µm thickness) were fixed to gelcoated microscope slides, and stained with hematoxylin and eosin. Topical features were measured using a Leica microscope (Leica Microsystems CMS GmbH, Leica Application Suite X, v.3.3.3.16958). Only villi that had a clearly defined luminal epithelial surface and submucosal muscle layer were measured. Similarly, only crypts with an easily visible submucosa cell layer and crypt wall inward pinch were measured. For statistical analysis, the mean value of each set of measurements was used (2-way ANOVA, Tukey’s and Sidak’s multiple comparison).

General lab equipment and chemicals, unless otherwise specified above, were purchased from Fisher Scientific.

## RESULTS

### Metagenomic profiles of humanization

**Figure 1.**
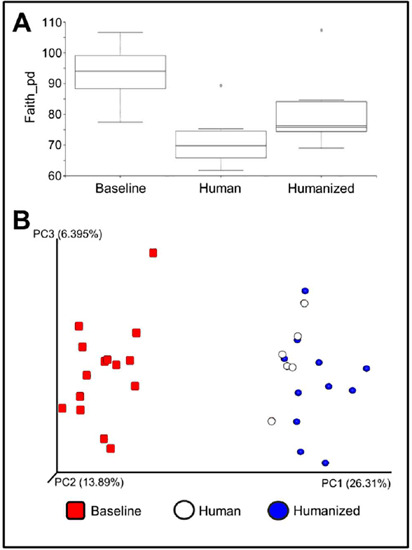
Humanization of rat gut microbiota. Both the (A) alpha-diversity (Faith’s phylogenetic diversity index) and (B) beta-diversity (weighted-normalized-Unifrac) of fecal bacterial communities of human donors and of Sprague Dawley rats before and after humanization, showing that the humanization of the gut significantly shifts the native rat microbiota profile toward the human counterpart.

Native gut microbiota of *naïve* Sprague Dawley rats were decimated prior to human fecal transplant, as previously implemented by our lab^22^. The stool samples from 15 *naïve* Sprague Dawley rats were collected before and after humanization and analyzed along with fecal material from the 5 human donors. Relative abundance and diversity of microbiota from rats and human donors was evaluated with 16S metagenomic analysis. Figure 1A compares alpha-diversity (Faith’s phylogenetic diversity metric) and illustrates the richness and diversity of the fecal microbiota before antibiotic treatment and humanization is significantly different from both the human and humanized samples (Kruskal-Wallis, pairwise, *p*=0.0018 and *p*=0.0020, respectively). Notably, the alpha-diversity of fecal microbiota is not significantly different between the samples from human donors and humanized rats. Beta-diversity between microbiota communities was evaluated using Bray-Curtis dissimilarity index and visualized by PCoA plot (Figure 1B). Significant dissimilarities can be easily discerned between the rat baseline/humanized and baseline/human samples (PERMANOVA, 999 permutations, *p*<0.001), while microbiota profiles of the human and humanized samples are more alike as they form a data cluster that can be easily separated from the rat baseline samples.

Table 1 lists the relative frequency of the detected phyla, where *Bacteroidetes* and *Firmicutes* accounted for 94%-95% of all identified phyla in rat, human and humanized rat samples. When comparing the *naïve* rat to the humanized rat, there was a significant decrease (*p*=0.001) in *Firmicute* populations. In the lesser phyla, humanization resulted in a significant reduction (*p*=0.029) of *Tenericutes*, and a downward trend in *Verrucomicrobia* and *Deferribacteres* populations (*p*=0.066 and *p*=0.91, respectively). The humanization process also resulted in a significant increase (*p*=0.002) in *Proteobacteria,* that surpassed human levels. This aberrant increase in *Proteobacteria* was also observed in our previous humanized model development study^22^. Additionally, *Actinobacteria* populations failed to expand closer to human levels even though *Actinobacteria* was detectable in the Sprague Dawley native microbiota *Fusobacteria*, which was present in only one of the human donor samples, was not established within the humanized rat.

**Table 1.** Average relative proportion (with standard deviation) of phyla detected in the fecal material of *naïve* Sprague Dawley rats (rat baseline), humanized rats (post-humanization baseline) and human donors. Significant changes between phyla of *naïve* rats and humanized rats were established by t-test (p<0.05).

	Human	Rat baseline	Humanized rat	p-value
Bacteroidetes	54.86%±7.28	51.02%±10.53	61.21%±5.82	0.291
Firmicutes	40.48%±8.58	44.52%±11.20	32.49%±6.27	0.001
Proteobacteria	2.91%±1.64	1.46%±0.94	6.14%±1.90	0.002
Actinobacteria	0.89%±0.73	0.04%±0.04	0.04%±0.05	0.395
Deferribacteres	0.00%±0.00	0.26%±0.05	0.02%±0.08	0.091
Tenericutes	0.01%±0.01	2.21%±2.44	0.00%±0.01	0.029
Verrucomicrobia	0.00%±0.00	0.45%±0.62	0.09%±0.14	0.066
Cyanobacteria	0.00%±0.00	0.04%±0.06	0.01%±0.03	0.125
Fusobacteria	0.86%±1.65	0.00%±0.00	0.00%±0.00	---

### Phylogenetic profling of microbiota populations following sleep fragmentation

**Supplementary Figure 1.**
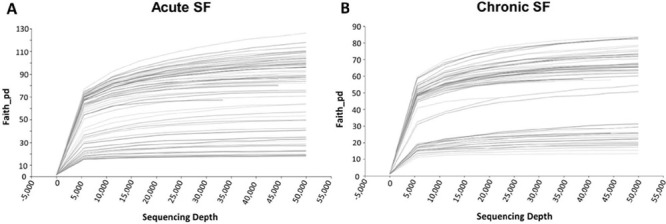
Rarefaction analysis of Sprague Dawley rats and humanized rats. Rarefaction analysis assessing species richness (Faith’s phylogenetic diversity) across all samples from (A) acute and (B) chronic sleep fragmentation studies.

Humanized rats were exposed to SF for either 6 days or 6 weeks, and gut microbiota communities were collected from the distal ileum, cecum and proximal colon for 16S rRNA analysis. Ion Torrent sequencing of the 136 samples collected after SF (acute: 10 SF subjects and 14 normal sleep subjects, with 23 distal ileum, 24 cecum, and 24 proximal colon samples collected; chronic: 11 SF subjects and 11 normal sleep subjects with 22 distal ileum, 22 cecum, and 21 distal ileum samples collected) resulted in 50,028,527 high-quality 250-bp single-end reads. A total of 8,249 and 8,053 features were identified in the acute and chronic SF analysis of 16S rRNA. Supplementary Figure 1 displays the rarefaction analysis (Faith’s phylogenetic index) for both acute and chronic SF studies. As noted in previous studies^10,34^, phylogenetic abundance and diversity in the distal ileum is reduced compared to microbiota populations of the cecum and proximal colon. However, a distinct separation of data is not evident in the acute study. This suggests that the humanization process may have encouraged an initial overgrowth of the transplanted microbiota in the small intestine. A similar change was observed in the small intestine following humanization in our previous study^22^. Based upon the results of this rarefaction analysis, alpha- and beta-diversity was profiled at a depth of 30,000 reads for maximum sample retention.

### Alpha and beta diversity are altered after acute and chronic sleep fragmentation

**Figure 2.**
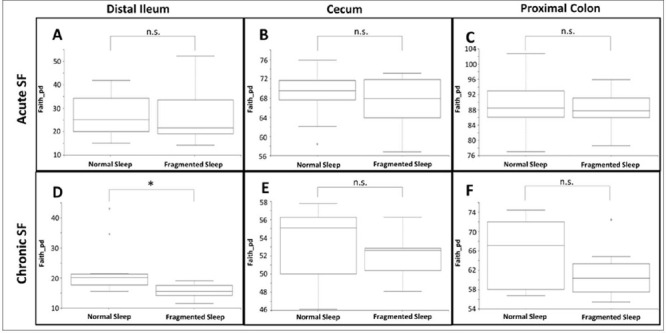
Altered alpha-diversity in microbiota of the distal ileum (A and D), cecum (B and E), or proximal colon (C and F), of humanized rats following acute (A, B, and C) or chronic (D, E, and F) sleep fragmentation (Faith’s phylogenetic diversity, Kruskal-Wallis).

Alterations to alpha-diversity richness in microbiota populations of the distal ileum, cecum and proximal colon after acute or chronic SF are shown in Figure 2. Following 6 days of SF, there were no statistically significant fuctuations in microbiota richness in any of the three intestinal regions (AC). However, a 6-week period of SF leads to a striking trend of decreased alpha-diversity in all sampled communities (DF), with a significantly diminished richness (Kruskal-Wallis (pairwise), *p*=0.0039) in the microbiota of the distal ileum (D).

**Figure 3.**
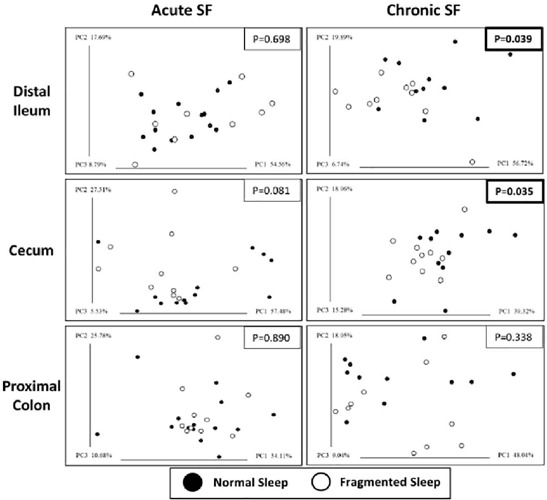
Chronic sleep fragmentation alters microbiota profiles. Beta diversity was measured using the weighted normalized Unifrac method and illustrated by PCoA plots. Significant alteration occurred in the distal ileum and cecum under chronic sleep fragmentation. The percent diversity captured (PC) by each axis is shown.

Changes in beta-diversity due to SF were measured using the weighted normalized Unifrac method. The result, visualized with PCoA plots (Figure 3), shows that chronic SF significantly altered beta-diversity in the distal ileum (PERMANOVA, *p*=0.039) and in the cecum (*p*=0.035), but not in the proximal colon. In contrast, acute SF did not result in a significant shift to beta-diversity in any region, although changes to cecal microbiota populations approached significance (*p*=0.081).

### Altered microbiota populations due to acute and chronic sleep fragmentation

**Figure 4.**
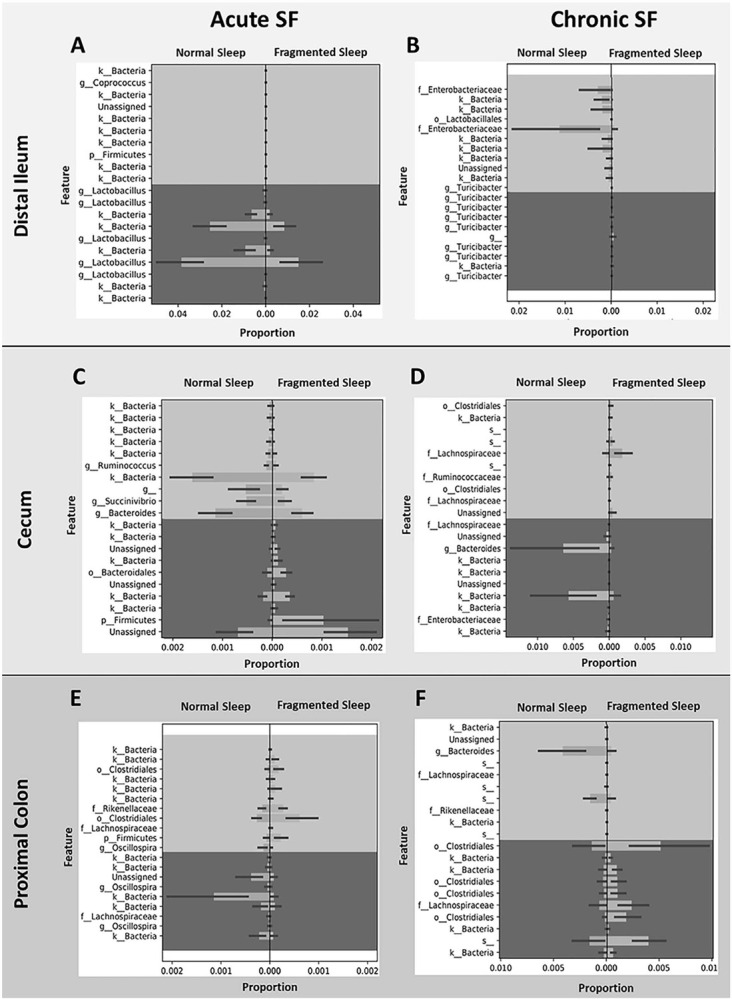
Gneiss balance profiles of microbiota alternation of humanized rats following sleep fragmentation. Listed are the top ten increased and decreased in microbial taxa in the distal ileum (A and B), cecum (C and D) and proximal colon (E and F) following either acute (A, C, and E) or chronic (B, D, and F) sleep fragmentation.

Gneiss balance profiling was used to identify significant changes to microbiota populations with acute or chronic SF compared to controls, and the results are presented in Figure 4. Gneiss detects abundance differentiation in microbial niches and infers changes in microbial subcommunities. The results showed multiple high-level balances, or shifts, in microbial niche communities. For example, in the distal ileum, acute SF (panel A) led to decreases vs. controls in multiple members of *Lactobacillus* (here, the genus is listed 5 separate times as the program was able to recognize, but not fully identify, species-level classifications). Also in the distal ileum, acute SF led to increases in *Firmicutes* and *Coprococcus*. Chronic SF (panel B) resulted in decreases vs. controls in the *Lactobacillales, Turicibacter,* and several members *of Enterobacteriaceae*, while multiple *Turicibacter* members expanded. In the cecum, acute SF (panel C) caused reductions in *Ruminococcus, Succinivibrio* and *Bacteroides,* and growth in the *Bacteroidales* and the *Firmicutes*, as compared to controls. Chronic SF (panel D) also resulted in reduced *Bacteroides, Enterobacteriaceae,* and *Lachnospiraceae*, while expansions occurred in *Ruminococcaceae* and multiple *Clostridiales* members, and *Lachnospiraceae* in the cecum when compared to controls.

The largest microbiota population changes in the proximal colon due to acute SF (panel E) include decreases vs. controls in multiple members of *Oscillospira* and increased vs. controls in *Firmicutes, Rikenellaceae*, and multiple *Clostridiales* members. Interestingly, *Lachnospiraceae* populations both expanded and contracted. This same pattern of *Lachnospiraceae* expansion and contraction was also observed in the proximal colon with chronic SF (panel F). In addition, multiple members of *Clostridiales* were increased, while *Bacteroides* and *Rikenellaceae* were reduced vs. controls after chronic SF.

### Prediction of metagenomic functions with PICRUSt analysis

PICRUSt and STAMP analyses were performed to infer functional consequences of SF-induced alterations to gut microbiota. Across all 3 intestinal regions in both the acute and chronic studies, 60 unique metabolic pathways were significantly (*p*<0.05) affected by SF-induced microbiota dysbiosis, with 7 pathways being altered under multiple conditions/in multiple regions. The majority of these pathways can be broadly classified into 10 functional categories (including nucleotide metabolism, DNA replication, repair & recombination, transcription & RNA processing, translation & protein processing, regulation of organismal systems, and metabolism of amino acids, carbohydrates, energy, lipids, terpenoids, polyketides, and xenobiotics). Most of these pathway changes were the result of perturbed microbial communities in the cecum, with 11 and 28 altered pathways identified in the acute and chronic SF studies, respectively. Table 2 summarizes the result of pathway alterations in each functional category following acute and chronic SF in each intestinal region.

**Table 2.** Summary of significant changes in the microbiota functions inferred by PICRUSt analysis. Listed are the numbers of significantly altered pathways in various functional categories due to microbiota dysbiosis in the distal ileum, cecum, and proximal colon of humanized rats after acute or chronic sleep fragmentation.

	Acute			Chronic			All	
	Distal ileum	Cecum	Proximal colon	Distal ileum	Cecum	Proximal colon	Acute	Chronic
Carbohydrate & energy metabolism	0	1	0	1	4	1	1	6
Lipid metabolism	0	1	0	1	0	1	1	2
Nucleotide metabolism	1	0	0	0	1	0	1	1
DNA replication, repair & recombination	0	0	0	0	6	0	0	6
Transcription & RNA processing	2	0	1	1	1	1	3	3
Amino acid metabolism	0	1	0	0	2	0	1	2
Translation & protein processing	1	2	0	0	5	0	3	5
Xenobiotics metabolism & degradation	1	1	0	0	0	1	2	1
Terpenoids & polyketides metabolism	0	3	2	2	3	1	5	6
Organismal systems	1	1	2	1	2	2	4	5
Organismal systems (cellular)	1	1	3	0	4	0	5	4
All	7	11	8	6	28	7	26	41

Of these functional categories, terpenoids and polyketides metabolism, organismal systems and organismal systems (cellular) contain the most pathway changes. Interestingly, the numbers of pathways affected by acute and chronic SF in most functional categories are very similar, with three exceptions, carbohydrate and energy metabolism, DNA replication, repair and recombination, and translation and protein processing (highlighted), whose perturbation mainly occurred in the cecum after chronic SF. Acute and chronic SF seem to target specific pathways concerning host functions; for instance, acute SF mainly affected the immune system in all three intestinal regions investigated, while chronic SF affected the excretory, endocrine, and nervous systems in a region-specific manner. The entire list of altered pathways in the distal ileum, cecum, and proximal colon following acute or chronic SF are shown in Supplementary Table 1.

### Consequences of sleep fragmentation upon microbial adhesion, penetration, and invasion

To determine if SF altered the capacity for bacteria to adhere and penetrate intestinal mucosal barriers or to undergo extraintestinal invasion of the mesentery, lymph nodes, spleen and liver, aerobic and anaerobic bacteria were cultured from these tissues. The results of the testing indicated that microbial invasion, adhesion, or penetration did not occur in any of the organs or intestinal regions tested (data not shown).

### Sleep fragmentation alters intestinal architecture

SF-induced changes to intestinal structures were assessed by measuring the length, width and number of crypts and villi, as well as the ratio of crypt to villi. No significant structural changes were observed in the distal ileum, cecum, or proximal colon for the acute study. However, chronic SF resulted in a significant decrease (*p*=0.021) in villus height in the proximal colon (318.9±35.4µm vs. 275.1±47.5µm), but not in the distal ileum (normal sleep=420.0±62.6µm; SF=431.1±73.0µm) or cecum (normal sleep=238.2±35.1µm; SF=255.0±33.5µm). On the other hand, the treatment did not cause significant change in the number of villi, crypt depth or crypt width in any intestinal location. Occludin staining analysis of intestinal tissues also did not show significant difference between normal sleep and SF samples (data not shown), suggesting that tight junction integrity was not affected by acute or chronic SF.

## DISCUSSION

Acute and chronic sleep fragmentation studies were conducted on Sprague Dawley rats harboring gut microbiota obtained from multiple human donors with diverse microbiota. A humanized model was necessitated, as fecal samples alone are not representative of the regional characteristics of gut microbiota; and utilization of the humanized model allowed for analysis of the contents and tissue samples in the intestinal regions and organs of interest in a coordinated manner, which ethically would not be feasible in human subjects. In this study, humanization of intestinal microbiota was accomplished in antibiotic-treated germ-free rats via repeated inoculations of human fecal material, a method successfully employed by ourselves and other investigators^22,35,36^.

Our approach in the development of this humanized model, which used fecal material sourced from multiple donors during fecal transplant, aided in creating a more complete and representative spectrum of human microbiota. Further, this approach can effectively eliminate donor-specific varieties, a confounding factor commonly associated with humanized rodent models; our “de-individualized” model will be extremely useful for mechanistic analysis of human gut microbiota modulation and the impact to the host systems.

The resulting humanized gut microbiota was distinctly more human-like than the pre-existing, native rodent gut microbiota, as evident by the results of the 16S rRNA gene sequencing and characterization. Perhaps not surprisingly, there was not a complete reconstitution of the human donor fecal microbiota, as antibiotic treatment does not completely (or permanently) eradicate all native rodent microbiota. Additionally, rat and human genetics, diet, and digestive tract physiologies are simply quite different. Furthermore, it is believed that human immune cells may play a role in microbiota stability and engraftment, and their functions might not be completely fulfilled by the rodent counterpart^37^. Interestingly, decimation of gut microbiota populations including *Tenericutes, Verrucomicrobia, Deferribacteres*, and *Cyanobacteria*, which was observed in rats post-humanization in this study, have generally been associated with overall improved health and positive effects. Conversely, the expansion of *Proteobacteria* is potentially worrisome as this abundant phylum is comprised of several known human pathogens and many species are associated with inflammation^38,39,40^. Despite the imperfect establishment of the human microbiota, our humanized rat model recapitulated gut microbiota that was substantially similar to the microbiota of the human fecal donors, as illustrated by the alpha- and beta-diversity studies.

Our data indicate that gut microbiota dysbiosis occurs after just 6 days of SF in humanized rats; and with chronic SF, aberrations to microbial populations increased. Acute SF tended to decrease the richness of alpha-diversity, though the changes were not statistically significant. Chronic SF resulted in a more drastic decline in the richness of microbiota in all three intestinal regions investigated, which reached statistical significance in distal ileum. In addition, chronic SF significantly shifted the beta-diversity of both the distal ileum and the cecum. Multiple taxa of gut microbiota were significantly altered with both acute and chronic SF. In a previous study of fecal pellets by Poroyko et al. (2016)^16^, sleep disruption in mice led to expansion of *Firmicutes, Lachnospiraceae, Ruminococcaceae*, and *Clostridiales*, while *Lactobacillaceae* populations contracted. Some of these changes (e.g., increases in *Oscillospira* and *Ruminococcus* of *Ruminococcaceae*) were also detected in a study of sleep-deprived rats using the ‘flowerpot’ method (which reduces REM sleep only), in addition to the changes in several other taxa (*Akkermansia, Parabacteroides, Phascolarctobacterium,* and *Aggregatibacter*)^41^. Similarly, the effect of SF treatment mirrored these trends in the study reported here. Specifically, acute SF resulted in increased *Firmicutes* in all three intestinal regions sampled.

*Firmicutes* are involved in energy resorption, and expansion of this phylum has been linked with obesity, low-grade inflammation, and subsequent development of chronic diseases^42,43,44^. One particular order of *Firmicutes* and *Clostridiales* was increased in both the cecum and proximal colon after SF. While some species of *Clostridiales* have been connected with overall mental health, other species are pathogenic and are associated with severe diarrhea, bowel diseases, and Parkinson’s disease^45,46,47^. Overgrowth of *Lachnospiraceae,* which occurred in the proximal colon after both acute and chronic SF, as well as in the cecum with chronic SF, is reportedly involved in intestinal inflammation and metabolic dysfunction^48,49^. In contrast, *Lactobacillus*, a genus of *Lactobacillaceae*, is well-recognized as beneficial lactic acid bacteria. *Lactobacillus*, which was decreased in distal ileum after SF in this study, exhibits a mutualistic relationship with humans, as it protects the host against potential invasions by pathogens. In fact, *Lactobacillus* is among the most commonly used probiotics that can maintain and/or improve human health. Interestingly, most of these changes were also observed in our previous study using native Sprague Dawley rats under the same experimental conditions^10^, although the models used in these two studies harboring gut microbiota of different origins, i.e., rat vs. human.

Detailed inspection of the results revealed that several taxa showed concordant changes; these include *Enterobacteriaceae* (decreased in distal ileum after chronic SF), *Ruminococcaceae* (increased in cecum after chronic SF), *Lachnospiraceae* (decreased in proximal colon after acute SF), and *Bacteroides* (decreased in proximal colon after chronic SF). Related taxa (at different taxonomic levels) showing the same change directions in response to SF were also detected, including *Lactobacillales/Lactobacillaceae/Lactobacillus* (decreased in distal ileum after acute and chronic SF), *Turicibacteraceae/Turicibacter* (increased in distal ileum after chronic SF), *Bacteroidales/Bacteroides* (increased in cecum after acute SF), and *Clostridiales/Clostridiaceae/Clostridium* (increased in cecum and proximal colon after chronic SF). As these changes frequently induced by SF in different models, they likely represent common targets of and/or common response to SF. Since SF results in changes in both beneficial (e.g., *Lactobacillus, Ruminococcaceae,* and *Bacteroides*) and pathogenic (*Lachnospiraceae, Enterobacter,* and *Clostridium*) taxa, understanding the functional impacts of these changes in an integrated manner will be essential for the intervention of the adverse effects of this stressor.

One caveat to note in this study is that while the age of the rats was the same at the beginning of SF (aged 13 weeks) by the end of the experiment the rats were of different ages (acute 14 weeks vs. chronic 19 weeks). This age difference may have had an impact on the microbiota present at the time of sampling. An alternative approach is to start SF late in the acute experiment so that the animals in the two experiments would be the same age at sample collection. However, this alternative merely changes the nature of the problem – an age difference at the start of SF treatment.

To obtain a clearer picture of how these SF-induced alterations to microbiota might affect the host, the functional impact of these changes was inferred using PICRUSt and STAMP analysis. Unsurprisingly, multiple metabolic pathways were altered, including metabolism of polyketides. Further, both acute and chronic SF led to significant decreases in the fundamental processes of transcription and translation. Alarmingly, chronic SF resulted in a diminished capacity for DNA repair, replication, and recombination. DNA repair, in particular, is vital for genome integrity, normal function, and ultimately, longevity and health. In fact, multiple genes initially associated with increased life span turned out to be involved in the repair of damaged DNA^50^. Comparison of this pathway list with that of our previous study^10^ revealed that SF induced unique changes in the humanized rat model, affecting the overall transcription and RNA processing activity of the gut microbiota, as well as the pathways essential for host functions, including glutamatergic synaptic transmission, cardiac muscle contraction, renin-angiotensin system, bicarbonate reclamation, and water absorption (vasopressin regulated). In contrast, pathways related to bacterial toxin synthesis and secretion that were activated in the native Sprague Dawley rats after SF^10^ were not detected in this study. Further targeted studies will be needed to verify these predictions and evaluate their biological significance and implications.

The mucosal barrier of the intestinal walls controls translocation of intestinal contents, protecting the rest of the body from exposure to pathogenic microorganisms and other toxins^51^. Previous studies have shown that total sleep deprivation increases bacterial translocation from the intestine to surrounding tissues and organs^15,52^. However, in this current study, SF did not change the ability of microbiota to adhere to or penetrate intestinal tissues, nor did it induce extraintestinal microbial invasion of the mesentery, lymph nodes, spleen, or liver. This result suggests that the gut does not become leaky even after the 6-week SF treatment employed in this study, which is consistent with that of our previous study^10^. This finding is further supported by the outcome of the occludin staining experiment that showed no significant changes in this protein, which suggests that the tight junction structure of the intestinal tract is relatively intact.

The intestines are highly specialized to acquire energy, serving as the main pathway of mineral and nutrient absorption. In addition, the intestines are also essential for metabolism, drug uptake, and immune response^53,54^. Intestinal walls are covered in self-renewing crypt-villi structures, which increase the absorptive surface area of the intestine and provide a quick response to physiological needs^55^. In the acute SF study, histological staining revealed no significant changes to intestinal architecture. However, chronic SF resulted in a significant decrease in villus height in the proximal colon, a morphological change suggestive of intestinal inflammation and villous atrophy that is associated with a variety of gastrointestinal problems.

There are several limitations in this study. For instance, the sleep architecture of the rats was not monitored during the SF treatments. Consequently, it would be difficult (if not impossible) to correlate the gut microbiota changes with the overall degree of sleep fragmentation, or with specific changes in different sleep stages. Despite this, the SF procedure employed in this study is highly effective. Ramesh et al. (2009)^23^ reported that this procedure significantly reduced both slow wave sleep and rapid eye movement sleep, based on EEG measurements. Furthermore, the treated animals also showed a significant decrease in wakefulness in the following dark (wakeful) period, with a significant increase in delta power of the EEG measurements. Considering the complexity of the humanization procedure, we reasoned that performing EEG measurements in these animals (which requires surgery) would likely introducing confounding factors in the data, which could be detrimental to the entire study.

We are also aware that the Greengenes database has not been updated since 2013. Thus, the taxonomic assignment of the features may not be up-to-date (e.g., many features in the Gneiss analysis are classified only at the level of bacteria). Furthermore, our data analysis pipeline could be further refined, which may produce a result with deeper insight into SF-associated gut microbiota change and its potential effect on the host.

In summary, both acute and chronic SF led to alterations in gut microbial populations and their collective effects on gut microbiota and host functions; and prolonged SF further resulted in increased microbiota dysbiosis and alterations to intestinal crypt/villi architecture. These findings provide insights into potential targets for preventive and/or therapeutic approaches aimed to maintain symbiotic homeostasis when confronted by the stress of disrupted sleep. Furthermore, the humanized model as described here will be useful for generating new knowledge concerning region-specific perturbation of gut microbiota that is highly relevant to human microbiome and health research.

## Appendix 2

**Supplementary Table 1.** Significant differences in the functional metagenome of microbiota predicted by PICRUSt analysis. List of all significantly altered metabolic pathways due to microbiota dysbiosis in the distal ileum, cecum, and proximal colon of humanized rats undergoing either acute or chronic sleep fragmentation. Seven pathways were affected by both acute and chronic SF and/or in multiple intestinal regions. White’s non-parametric t-test with Benjamini-Hochberg FDR post hoc test was used in determining statistical significance (p<0.05).

	KEGG pathway	Sleep fragmentation	Intestinal region	p-value	Change in mean proportions (%)
A	**Carbohydrate & energy metabolism**				
1	Glycolysis / Gluconeogenesis	Acute	Cecum	0.033	-0.0250
2	Ascorbate and aldarate metabolism	Chronic	Distal Ileum	0.015	-0.0220
3	Carbohydrate metabolism	Chronic	Cecum	0.014	0.0210
4	Carbon fixation pathways in prokaryotes	Chronic	Cecum	0.044	-0.0520
5	Citrate cycle	Chronic	Cecum	0.043	-0.0830
6	Oxidative phosphorylation	Chronic	Cecum	0.023	-0.1370
7	Inositol phosphate metabolism	Chronic	Proximal colon	0.039	-0.0070
B	**Lipid metabolism**				
8	Lipid biosynthesis proteins	Acute	Cecum	0.042	-0.0200
9	Steroid biosynthesis	Chronic	Distal ileum	0.049	0.0001
10	Linoleic acid metabolism	Chronic	Proximal colon	0.042	-0.0050
C	**Nucleotide metabolism**				
1	Purine metabolism	Acute	Distal ileum	0.042	-0.1880
2	Pyrimidine metabolism	Chronic	Cecum	0.012	-0.0600
D	**DNA replication, repair & recombination**				
1	DNA replication proteins	Chronic	Cecum	0.015	-0.0510
2	DNA replication	Chronic	Cecum	0.011	-0.0330
3	DNA repair and recombination proteins	Chronic	Cecum	0.007	-0.1030
4	Mismatch repair	Chronic	Cecum	0.042	-0.0270
5	Nucleotide excision repair	Chronic	Cecum	0.014	-0.0150
6	Homologous recombination	Chronic	Cecum	0.024	-0.0400
E	Transcription & RNA processing				
1	RNA polymerase	Acute	Distal ileum	0.048	-0.0230
2	Transcription related proteins	Acute	Distal ileum	0.050	-0.0120
3	Transcription machinery	Acute	Proximal colon	0.004	-0.0310
4	RNA degradation	Chronic	Distal ileum	0.030	0.0110
5	RNA polymerase	Chronic	Cecum	0.019	-0.0170
6	RNA polymerase	Chronic	Proximal colon	0.021	-0.0110
F	**Amino acid metabolism**				
1	Tyrosine metabolism	Acute	Cecum	0.021	-0.0130
2	Phenylalanine, tyrosine and tryptophan biosynthesis	Chronic	Cecum	0.032	0.0240
3	Amino acid related enzymes	Chronic	Cecum	0.009	-0.0450
G	**Translation & protein processing**				
1	Glycosyltransferases	Acute	Distal ileum	0.027	-0.0520
2	Ribosome biogenesis in eukaryotes	Acute	Cecum	0.028	-0.0020
3	Protein processing in endoplasmic reticulum	Acute	Cecum	0.037	-0.0060
4	Ribosome	Chronic	Cecum	0.008	-0.1500
5	Ribosome biogenesis in eukaryotes	Chronic	Cecum	0.042	-0.0020
6	Translation factors	Chronic	Cecum	0.011	-0.0270
7	Protein export	Chronic	Cecum	0.027	-0.0490
8	Peptidoglycan biosynthesis	Chronic	Cecum	0.026	-0.0270
H	**Xenobiotics metabolism & degradation**				
1	Drug metabolism - other enzymes	Acute	Distal ileum	0.040	0.0110
2	Atrazine degradation	Acute	Cecum	0.034	0.0080
3	Bisphenol degradation	Chronic	Proximal colon	0.049	-0.0060
I	**Terpenoids & polyketides (& secondary metabolites) metabolism**				
1	Geraniol degradation	Acute	Cecum	0.049	-0.0040
2	Flavonoid biosynthesis	Acute	Cecum	0.035	0.0030
3	D-Arginine and D-ornithine metabolism	Acute	Cecum	0.033	0.0030
4	Biosynthesis of type II polyketide products	Acute	Proximal colon	0.031	-0.0001
5	Indole alkaloid biosynthesis	Acute	Proximal colon	0.016	-0.0001
6	Carotenoid biosynthesis	Chronic	Distal ileum	0.039	0.0010
7	**Tetracycline biosynthesis**	Chronic	Distal ileum	0.013	0.0110
8	Prenyltransferases	Chronic	Cecum	0.039	-0.0270
9	Terpenoid backbone biosynthesis	Chronic	Cecum	0.016	-0.0310
10	Zeatin biosynthesis	Chronic	Cecum	0.025	-0.0050
11	Vitamin B6 metabolism	Chronic	Proximal colon	0.040	0.0080
J	Organismal systems				
1	RIG-I-like receptor signaling pathway	Acute	Distal ileum	0.015	-0.0020
2	NOD-like receptor signaling pathway	Acute	Cecum	0.048	-0.0020
3	Bacterial invasion of epithelial cells	Acute	Proximal colon	0.038	-0.0001
4	Cardiac muscle contraction	Acute	Proximal colon	0.039	-0.0010
5	Vasopressin-regulated water reabsorption	Chronic	Distal ileum	0.026	0.0001
6	Glutamatergic synapse	Chronic	Cecum	0.050	-0.0070
7	Cardiac muscle contraction	Chronic	Cecum	0.015	-0.0130
8	Renin-angiotensin system	Chronic	Proximal colon	0.020	0.0001
9	Proximal tubule bicarbonate reclamation	Chronic	Proximal colon	0.009	0.0040
K	**Organismal systems (cellular)**				
1	Apoptosis	Acute	Distal ileum	0.048	-0.0060
2	Phosphatidylinositol signaling system	Acute	Cecum	0.033	-0.0040
3	Notch signaling pathway	Acute	Proximal colon	0.031	-0.0001
4	Wnt signaling pathway	Acute	Proximal colon	0.031	-0.0001
5	p53 signaling pathway	Acute	Proximal colon	0.049	-0.0001
6	Phosphatidylinositol signaling system	Chronic	Cecum	0.046	-0.0090
7	Apoptosis	Chronic	Cecum	0.017	-0.0040
8	p53 signaling pathway	Chronic	Cecum	0.014	-0.0040
9	Cell cycle – Caulobacter	Chronic	Cecum	0.018	-0.0260
